# Dissociating the neural correlates of tactile temporal order and simultaneity judgements

**DOI:** 10.1038/srep23323

**Published:** 2016-04-11

**Authors:** Makoto Miyazaki, Hiroshi Kadota, Kozue S. Matsuzaki, Shigeki Takeuchi, Hirofumi Sekiguchi, Takuo Aoyama, Takanori Kochiyama

**Affiliations:** 1 Department of Computer Science, Faculty of Informatics, Shizuoka University, 3-5-1 Johoku, Naka-ku, Hamamatsu, shizuoka 432-8011, Japan; 2 Research Institute, Kochi University of Technology, 185 Miyanokuchi, Tosayamada, Kami-city, Kochi 782-8502, Japan; 3 Faculty of Business and Information Sciences, Jobu University, 634-1 Toyazukamachi, Isesaki, Gumma 372-8588, Japan; 4 Research Institute for Time Studies, Yamaguchi University, 1677-1 Yoshida, Yamaguchi 753-8511, Japan; 5 ATR Brain Activity Imaging Center, 2-2-2 Hikaridai, Seika-cho, Sorakugun, Kyoto 619-0288, Japan

## Abstract

Perceiving temporal relationships between sensory events is a key process for recognising dynamic environments. Temporal order judgement (TOJ) and simultaneity judgement (SJ) are used for probing this perceptual process. TOJ and SJ exhibit identical psychometric parameters. However, there is accumulating psychophysical evidence that distinguishes TOJ from SJ. Some studies have proposed that the perceptual processes for SJ (e.g., detecting successive/simultaneity) are also included in TOJ, whereas TOJ requires more processes (e.g., determination of the temporal order). Other studies have proposed two independent processes for TOJ and SJ. To identify differences in the neural activity associated with TOJ versus SJ, we performed functional magnetic resonance imaging of participants during TOJ and SJ with identical tactile stimuli. TOJ-specific activity was observed in multiple regions (e.g., left ventral and bilateral dorsal premotor cortices and left posterior parietal cortex) that overlap the general temporal prediction network for perception and motor systems. SJ-specific activation was observed only in the posterior insular cortex. Our results suggest that TOJ requires more processes than SJ and that both TOJ and SJ implement specific process components. The neural differences between TOJ and SJ thus combine features described in previous psychophysical hypotheses that proposed different mechanisms.

We live in a dynamic world that changes over time. Temporal relationships amongst sensory events provide key information for recognition of the world. The psychophysics of temporal order judgement (TOJ) of sensory events has been widely investigated^1^,^2^,^3^,^4^,^5^,^6^. In TOJ, participants receive two stimuli with a certain stimulus onset asynchrony (SOA) and judge which stimulus is presented first (or last). The judgement proportion of TOJ as a function of the SOA typically exhibits a sigmoidal curve that can be fit to a cumulative Gaussian function. Thus, the psychometric function provides two parameters: the mean and the standard deviation (σ). The mean represents the point of subjective simultaneity (PSS), and σ represents the perceptual time sensitivity.

The TOJ paradigm has revealed various psychophysical phenomena. For example, the prior-entry effect (i.e., the effect of attention on perceptual speed) has been demonstrated using TOJs^5^,^7^. In another example, the crossing of arms increases the rate of misreporting of tactile TOJ across the hands^6^,^8^,^9^,^10^. This crossed-arm deficit suggests that the brain processes the spatial locations of the hands before temporally ordering the tactile signals from the respective hands.

In another popular task, simultaneity judgement (SJ), participants judge whether two stimuli are presented simultaneously or successively. The judgement proportion of SJ exhibits a bell-shaped curve that can be fit to a Gaussian frequency function. Thus, similar to TOJ, SJ also provides the mean (PSS) and the standard deviation (σ).

The SJ paradigm has also been used for probing psychophysical phenomena. For example, perceptual adaptation to a coherent lag between audio and visual stimuli has been identified using SJ^11^. This lag adaptation effect has also been observed in audiovisual^4^,^12^,^13^,^14^,^15^ and other cross-modal^16^,^17^ TOJs. Accordingly, the experimental results of TOJ and SJ have sometimes been discussed in the context of the assumption that both tasks reflect the same psychological or neural mechanisms^4^,^15^,^18^. Some classical studies assumed that the perception of successiveness is a necessary and sufficient condition for the correct perception of temporal order, in which the perceptual latency model^19^ assumed that TOJ and SJ are based on the same internal events (reviewed in Allan^20^).

However, some studies have argued that the perception of successiveness/simultaneity is a necessary but not sufficient condition for the perception of temporal order^2^,^21^ (see also ref. 3). This conditional relationship suggests that the perceptual processes for SJ are included in those for TOJ, but TOJ requires more processes. More specifically, when performing TOJ, the brain further operates the process to identify or determine the order of two stimuli after detecting or comparing the successiveness/simultaneity of the stimuli. This argument is based on the observation that, compared to SJs, TOJs require longer SOAs for correct judgements (i.e., exhibit larger σ values). Similar results have been observed in audio^22^, visual^23^, and tactile^24^ tasks. Moreover, some psychophysical effects dominantly or specifically occur in TOJ. For example, selective attention has a smaller effect on SJ than it does on TOJ^25^,^26^. In addition, the crossed-arm deficit observed in tactile TOJ^6^,^8^,^9^,^10^ does not occur in tactile SJ^24^,^27^. The TOJ-specific effects therefore suggest the presence of TOJ-specific processes in the brain. Consequently, psychophysical studies are forming a consensus that TOJ involves more processes than SJ^2^,^21^,^24^,^25^,^28^,^29^.

In the present study, we performed functional magnetic resonance imaging (fMRI) in participants engaged in TOJ and SJ with identical tactile stimuli to identify differences in neural activity during the two tasks. One aim of our study was to test the psychophysical hypothesis that TOJ involves more processes than SJ by directly comparing the neural correlates of TOJ and SJ. If the hypothesis is valid, TOJ should activate more brain regions than SJ. Based on a recent fMRI study^30^, we developed working hypotheses for two possible TOJ-specific regions: (1) the ventral attention network and (2) the motor control network.

Takahashi *et al*.^30^ reported neural correlates of tactile TOJ using tactile number judgement as a control task. In their report, the authors noted that the TOJ-related regions overlapped with the ventral attention network, which consists of the temporal parietal junction (TPJ) and ventral frontal cortices (VFC); this network functions to detect salient or unexpected stimuli^31^. Within the ventral attention network, the authors remarked that the TPJ is essential for not only attention but also TOJ. In addition, we focused on their results showing that the TOJ-related regions also overlapped with regions that participate in motor control^32^,^33^,^34^,^35^, such as the premotor cortex (PMC), posterior parietal cortex (PPC), and cerebellum. Notably, a recent psychophysical study reported that when participants planned to cross their arms, the crossed-arm deficit^6^,^8^,^9^,^10^ occurred even when TOJ was performed with *un*crossed arms^1^. Thus, motor planning or prediction is reflected in TOJ processing, suggesting that motor control and TOJ may share the same neural mechanism.

The second aim of our study was focused on the presence or absence of SJ-specific activity. According to the above assumption that the perception of successiveness/simultaneity is a necessary but not a sufficient condition for perception of temporal order, the perceptual processes for SJ are included in those for TOJ. That is, the conditional relationship also provides an additional hypothesis that SJ induces no specific activity compared to TOJ. Indeed, compared to TOJ, there is less evidence for psychophysical effects specific to SJ. Nevertheless, some theoretical models^3^,^23^,^36^ have proposed independent paths for TOJ and SJ. Some evidence even suggests an SJ-specific psychophysical effect^37^. We cannot exclude the possibility that the human brain also implements an SJ-specific process. If SJ-specific activity were found in the present study, the finding would provide neural evidence for the presence of an SJ-specific process in the brain.

## Results

**Figure 1:**
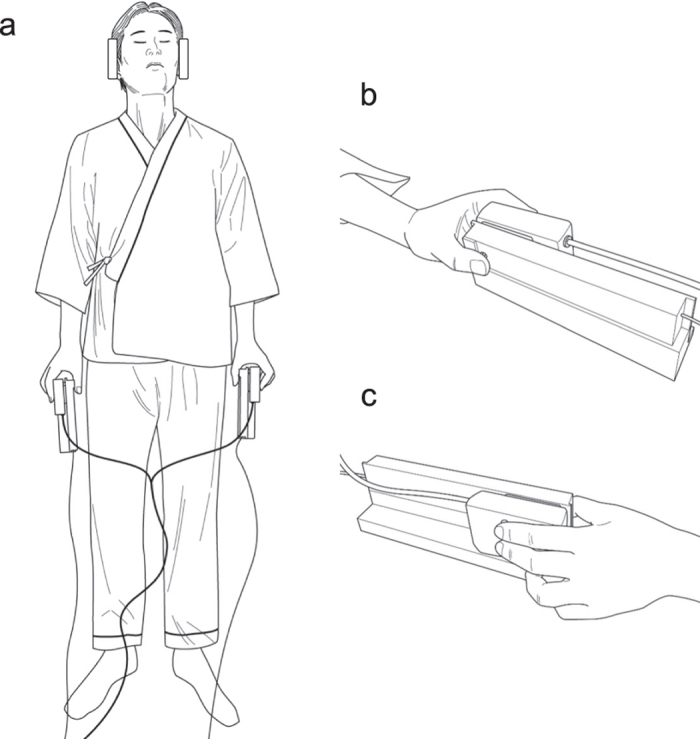
Overview of the experimental setup inside the MRI scanner. (**a**) A top view of a participant holding a pair of stimulus-response devices. (**b**) A palm-side view of a hand grasping the device. (**c**) A back-side view of a hand grasping the device.

During fMRI scanning, 16 participants performed TOJ and SJ for two tactile stimuli, one stimulus delivered to each hand, with closed eyes (Fig. 1). SOAs were pseudo-randomly sampled at 11 intervals (−180, −90, −30, −10, −5, 0, +5, +10, +30, +90, and +180 ms, in which positive values signify “right precedes left”). We used a block design for the fMRI scanning. Within each block (30 s), all 11 SOAs were presented (i.e., 11 trials/block), and the participants performed one of the two tasks. The TOJ and SJ blocks were alternated, and each block was followed by a rest period (15 s). Each participant performed 4 sessions (8 blocks/session). That is, each participant completed 352 trials in total (176 trials for each task) during the scanning.

### Behavioural results

**Figure 2:**
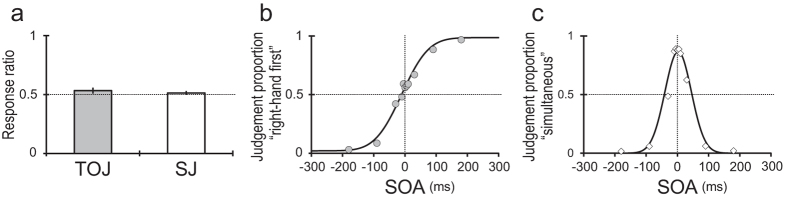
Behavioural data across participants (*N* = 16). (**a**) The response ratio was 0.53 ± 0.03 (mean ± SEM) for TOJ (“right-hand first”) and 0.51 ± 0.02 for SJ (“simultaneous”). This difference was not significant (*P* = 0.53). (**b**) The proportion of the trials judged as “right-hand first” during TOJ as a function of SOA. (**c**) The proportion of the trials judged as “simultaneous” during SJ as a function of SOA. The PSS and σ values for TOJ and SJ were calculated from the psychometric functions of each participant. The PSS was −8.71 ± 6.62 ms for TOJ and 2.34 ± 1.36 ms for SJ, which were not significantly different (*P* = 0.17). The σ was 59.81 ± 7.53 ms for TOJ and 36.12 ± 5.79 ms for SJ, which were significantly different (*P* < 0.001).

The response ratio across the participants was 0.53 ± 0.03 (mean ± SEM) for the “right-hand first” judgement in TOJ and 0.51 ± 0.02 for the “simultaneous” judgement in SJ (Fig. 2a). There were no differences between the tasks (*P* = 0.53). Thus, sensory inputs and motor output ratios were balanced between TOJ and SJ across the blocks.

Figures 2b,c show the proportion of trials in which the participants judged “right-hand first” for TOJ and “simultaneous” for SJ as a function of SOA. The psychometric functions exhibited a typical sigmoidal curve for TOJ (Fig. 2b) and a bell-shaped curve for SJ (Fig. 2c). The PSS across the participants was −8.71 ± 6.62 ms for TOJ and 2.34 ± 1.36 ms for SJ, which were not significantly different (*P* = 0.17). However, the σ across the participants was significantly greater for TOJ (59.81 ± 7.53 ms) than for SJ (36.12 ± 5.79 ms; *P* < 0.001).

Notably, the greater σ values (i.e., less perceptual time sensitivity) for TOJ than for SJ were consistent with the psychophysical observations in previous studies using tactile^24^, audio^22^, and visual^23^ stimuli. Thus, the difference in time sensitivity is a necessary phenomenon occurring between monomodal TOJ vs. SJ.

### fMRI results

**Figure 3:**
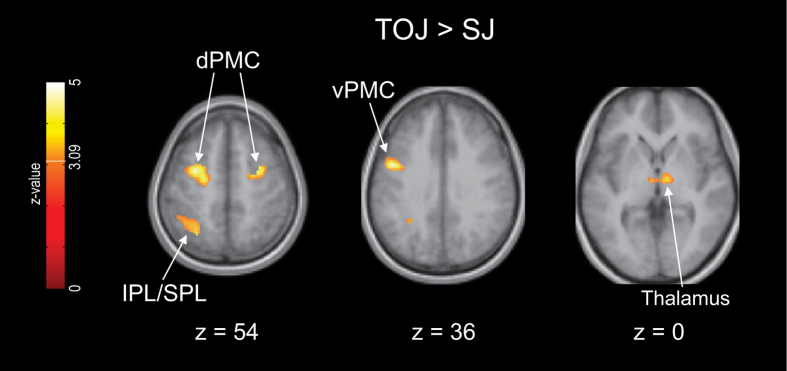
Brain regions that were more strongly activated during TOJ than during SJ. The results are from a 16-participant group analysis. Significantly activated voxels were identified using a significance threshold of *P* < 0.001 uncorrected at the voxel level (*Z* = 3.09) and *P* < 0.05 FWE-corrected at the cluster level. The TOJ > SJ contrasts were inclusively masked with the TOJ > rest contrasts (*P* < 0.05 uncorrected at the voxel level). In this contrast, the σ difference between the tasks was regressed out as a covariate of no interest. Furthermore, the voxels that correlated with the σ differences (*P* < 0.05 uncorrected at the voxel level) were exclusively masked. dPMC, dorsal premotor cortex; vPMC, ventral premotor cortex; IPL, inferior parietal lobule; SPL, superior parietal lobule.

**Figure 4:**
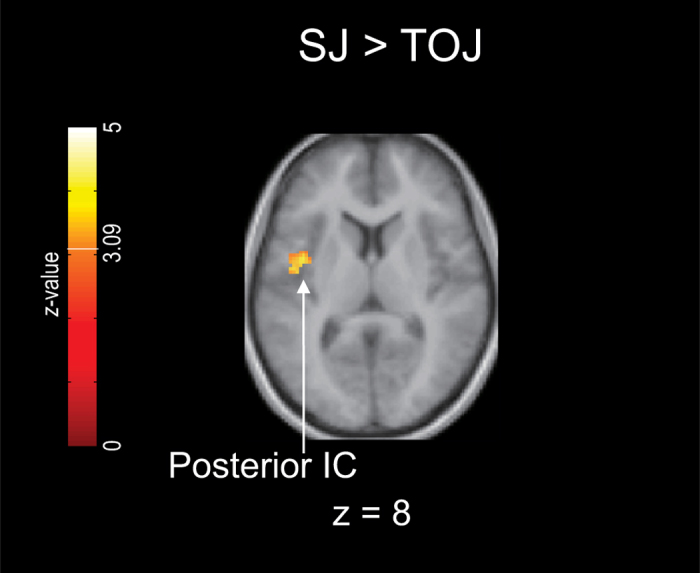
Brain regions that were more strongly activated during SJ than during TOJ. The results are from a 16-participant group analysis. Significantly activated voxels were identified using a significance threshold of *P* < 0.001 uncorrected at the voxel level (*Z* = 3.09) and *P* < 0.05 FWE-corrected at the cluster level. The SJ > TOJ contrasts were inclusively masked with the SJ > rest contrasts (*P* < 0.05 uncorrected at the voxel level). In this contrast, the σ difference between the tasks was regressed out as a covariate of no interest. Furthermore, the voxels that correlated with the σ differences (*P* < 0.05 uncorrected at the voxel level) were exclusively masked. IC, insular cortex.

Figures 3 and 4 show the contrasts between the regions activated during TOJ and SJ (TOJ > SJ and SJ > TOJ, details in Supplementary Tables S1 and S2 online). In these contrasts, we excluded the effect of the difference in σ between TOJ and SJ (see *fMRI data analysis* in Methods) based on the standard interpretation^38^ and adjustment procedure^39^,^40^ for the difference in difficulty between the tasks in the fMRI studies. We used a significance threshold of *P* < 0.001 uncorrected at the voxel level and *P* < 0.05 FWE (family-wise error) corrected at the cluster level^41^.

The TOJ > SJ contrast exhibited greater activation of the left dorsal premotor cortex (dPMC), the left ventral premotor cortex (vPMC), the right dPMC, left PPC (inferior parietal lobule, IPL, and superior parietal lobule, SPL), and the bilateral thalamus (Fig. 3, clusters 1–5 in Table S1). The activation cluster of the PPC extended to the marginal area with the superior temporal gyrus (STG) (cluster 4 in Table S1). In addition, when moderating the significant threshold at the cluster level (*P* < 0.05 uncorrected) without changing that at the voxel level (*P* < 0.001 uncorrected), the TOJ > SJ contrast exhibited activation in the right cerebellum (clusters 6 and 8 in Table S1). Moreover, the right cerebellum revealed significant activation in the TOJ > rest contrast but not in the SJ > rest contrast (Supplementary Fig. S1 and Tables S3 and S4 online).

Meanwhile, the SJ > TOJ contrast revealed greater activation only in the left posterior insular cortex (IC) (Fig. 4, cluster 1 in Table S2). Using the liberal threshold at the cluster level (*P* < 0.05 uncorrected), we also observed greater activation in the right posterior IC (cluster 2 in Table S2).

## Discussion

In our fMRI study, the TOJ > SJ contrast revealed stronger activation in the left vPMC, the bilateral dPMC, the left PPC, and the bilateral thalamus. The TOJ-specific activation was thus observed in multiple brain regions, whereas the SJ > TOJ contrast revealed stronger activation in the posterior IC *only*. Therefore, regarding the first aim of the present study, our fMRI study supported the psychophysical hypothesis that TOJ involves more processes than SJ^2^,^21^,^24^,^25^,^28^,^29^. Regarding the second aim of the present study, our results thus suggest that not only TOJ but also SJ involves its own specific processes. Some psychophysical studies have proposed that TOJ requires more processes than SJ, whereas the processes for SJ are included in those for TOJ^2^,^21^,^25^,^28^. Other studies have proposed two independent processes for TOJ and SJ^3^,^23^,^36^. Our fMRI results thus exhibit combined features of these two psychophysical proposals. That is, the brain implements independent processes for TOJ (e.g., determination of temporal order) and SJ (e.g., detection of simultaneity/successiveness); however, the brain recruits more regions for TOJ-specific processing. We subsequently discuss the task-specific regions.

### TOJ-specific regions

Based on a recent fMRI study^30^, we developed two working hypotheses for the possible TOJ-specific regions: (1) the ventral attention network and (2) the motor control network. First, the ventral attention network consists of the right TPJ and VFC^31^. In our TOJ > SJ contrast, activation of the PPC extended to the marginal area with the STG (cluster 4 in Table S1), suggesting that the TOJ-specific region overlapped with the TPJ. In addition, using the liberal threshold at the cluster level (*P* < 0.05 uncorrected), the TOJ > SJ contrast exhibited peak activity in the left anterior IC (cluster 7 in Table S1). These observations suggested that a region near the VFC or a part of the VFC may have been activated. However, these activations were observed in the left hemisphere. The ventral attention network is considered to be lateralised to the right hemisphere^31^. Thus, the fMRI results do not support our first working hypothesis.

Second, most of the TOJ-specific regions detected in the present study overlap with the regions that participate in motor control^32^,^33^,^34^,^35^. In the TOJ > SJ contrast, greater activation was observed in the left vPMC, the bilateral dPMC, and the left PPC. Using the liberal threshold at the cluster level, greater activation also appeared in the right cerebellum, which exceeded the threshold of the FWE correction for the TOJ > rest contrast but not for the SJ > rest contrast. In addition, the TOJ > SJ contrast exhibited significant activation in the thalamus bilaterally. Thalamus activity was also observed in a previous fMRI study on tactile TOJ using a time-irrespective control task^30^, and the thalamus plays a role in generating movements and monitoring one’s own actions^42^,^43^,^44^. These observations favour our second working hypothesis that the motor control network is activated specifically by TOJs. Notably, the TOJ-specific cerebral activations were primarily distributed in the left hemisphere even though the right vs. left hand factor was counterbalanced in our study (see *Tasks* in Methods). Based on the consistent involvement of the left premotor and parietal cortices in perceptual temporal expectations, Coull and Nobre^45^ proposed a general temporal prediction network for perceptual and motor states. Our observation suggests that tactile TOJ involves the general temporal prediction network.

The TOJ-specific regions observed in the present study are consistent with a recent psychophysical finding that the crossed-arm deficit occurs in TOJ with *un*crossed arms when the participants planned to cross their arms^1^. The predictive crossed-arm deficit suggests that the process required for motor control interacts with that for the temporal ordering of tactile stimuli, which is in accordance with our fMRI results indicating that the motor control-related regions were activated during tactile TOJ. In the sensorimotor system, ordering the sequence of body movements is essential for performing skilled motor actions^46^,^47^. It is reasonable that identifying or determining the temporal order of somatosensory signals and controlling motor actions share neural correlates in the sensorimotor system.

Moreover, our fMRI results also explain the occurrence of the crossed-arm deficit observed during TOJs^6^,^8^,^9^,^10^ but not during SJs^24^,^27^. Wada *et al*.^48^ recently demonstrated that fMRI activity in the left PPC increased when participants crossed their arms and that activity in this area was positively correlated with the magnitude of the crossed-arm deficit. The left PPC was also activated in our TOJ > SJ contrast but not in our SJ > TOJ contrast. The lack of a crossed-arm deficit during SJs is in agreement with our present observation of less activity in the left PPC during SJ than during TOJ.

### SJ-specific region

In the present study, the posterior IC was revealed to be an SJ-specific region. The IC has been identified as a neural correlate for human temporal processing^49^ although this role has only been reported for the anterior IC. A recent fMRI study reported posterior IC activation during a time reproduction task^50^. Using a time reproduction task with long tone intervals (9 and 18 s), Wittmann *et al*.^50^ found that the posterior IC exhibited activation during the encoding phase, whereas the anterior IC was activated during the reproduction phase. The IC is also a known neural correlate for interoception. The posterior IC represents primary interoceptive information, whereas the anterior IC is associated with the later or higher stage of emotional evaluations regarding interoceptive feelings^51^. Thus, the posterior IC is engaged in early perceptual processing instead of later decisional or motor processing. Based on these findings, we suggest that the posterior IC should be involved in the perceptual stage of SJ.

A previous fMRI study^52^ identified the right parietal IC as one of the neural correlates of visual SJ compared to visual orientation judgement. Although the peak activation of the parietal IC (Table 2 in Lux *et al*.^52^) was located more posterior to the IC activation in our SJ > TOJ contrast, Fig. 3b in Lux *et al*. shows that the parietal IC activation includes a relatively anterior area, suggesting that visual SJ also activates a region similar to the posterior IC in our SJ > TOJ contrast. Along with the parietal IC, Lux *et al*. also detected neural correlates for visual SJ in other regions, such as the left TPJ, the left inferior frontal gyrus (IFG), the left middle frontal gyrus, and the left STG. These regions should contain the regions associated with general temporal processing that is not always specific to SJ. Our observations suggest that the posterior IC is involved in SJ-specific process components, such as detectors^3^,^23^ or comparators^36^, for judgements of simultaneity/successiveness of tactile stimuli.

### Regions possibly related to differences in task difficulty

In the contrasts between the tasks, we excluded the effect of the σ difference according to previous reports indicating that differences in difficulty between the tasks should be regressed out in the brain imaging contrasts^39^,^40^. However, σ values that are higher for TOJ than for SJ are consistent with previous psychophysical observations^22^,^23^,^24^, which is one of grounds for the hypothesis that TOJ involves more processes than SJ. Namely, the exclusion of the effect of the σ difference may exclude the essential regions of interest in our study. Without exclusively masking the voxels that were correlated with the σ difference, similar results were obtained, except that the activated voxel size (*k*_*E*_) of the bilateral pre-supplementary motor areas (pre-SMAs) (*k*_*E*_ = 104; *P* = 0.030 uncorrected; cluster 9 in Table S1) increased (*k*_*E*_ = 170; *P* = 0.008 uncorrected) for the TOJ > SJ contrast. This observation is consistent with a previous report indicating that the bilateral pre-SMAs showed difficulty-dependent activity during a duration discrimination task^38^. Thus, the pre-SMAs may be a TOJ-specific region, if we abandon the convention that neural correlates of task difficulty should not be recognised as task-related regions.

### Perspectives for future studies: commonalities or differences amongst tasks and sensory modalities

In the present study, we primarily aimed to identify differences in neural activity between TOJ and SJ. To fully clarify the relationships between TOJ and SJ in the future, it will be necessary to directly identify the neural correlates of the common temporal processing components for TOJ and SJ. Although the common neural correlates for both tasks could not be fully separated from response-related activity in our present study, the basal ganglia (BG) may be a putative common component for temporal processing. In our study, the BG was activated during both TOJ and SJ compared to the baseline resting periods (clusters 2 and 3 in Supplementary Table S5 online). Takahashi *et al*.^30^ also reported TOJ-related activity in the BG using a time-irrelevant control task. The BG is considered to be a core component of perceptual interval timing^45^,^53^. We speculate that the BG represents time intervals between two tactile stimuli. Based on the intervals, the posterior IC discriminates between simultaneous and successive stimuli, and multiple regions, such as the PMCs and the PPC, determine the temporal order of the tactile stimuli.

The differences or commonalities amongst sensory modalities should also be addressed in future studies. For example, the frontal eye field (FEF) was found to be one of the neural correlates in visual TOJ^54^ but not in visual SJ^52^. In the visual modality, the TOJ > SJ contrast may reveal FEF activity, suggesting that visual TOJ shares neural correlates with the control of eye movements. This hypothesis may be supported by psychophysical findings of interactions between visual TOJs and eye movements^55^,^56^.

Laterality may be a significant issue. In our study using tactile stimuli, the TOJ-specific regions were primarily distributed in the left hemisphere. A lesion study using auditory TOJ suggested a left hemisphere dominance in temporal order processing^57^, which is consistent with an fMRI study on syllable order judgement^58^. In vision, however, a number of lesion studies have suggested that the right hemisphere (particularly the PPC/IPL) plays a dominant role in visual temporal order processing^59^,^60^. Similar differences in laterality may be found in SJs. Our tactile SJ activated the posterior IC more reliably in the left hemisphere, whereas visual SJ activated only the right parietal IC^52^.

However, there are arguments that the right hemisphere is not involved specifically in temporal processing but is instead involved in attention^54^,^57^. Indeed, lesion studies involving visual TOJs have been advanced in the context of the prior entry effect^60^, namely, the effect of attention on perceptual speed. Davis *et al*.^54^ emphasised the left TPJ (including PPC) as an important area for visual TOJ. Moreover, in our TOJ > SJ contrast, activation of the left PPC extended to the boundary with the STG. This observation may suggest that the left TPJ is involved in TOJ, independent of differences in the sensory modality. Thus far, the results and interpretations of the laterality of temporal order processing are complicated. In addition to the accumulation of neural evidence obtained with whole-brain imaging (e.g., fMRI), further investigations using transient disruption techniques, such as transcranial magnetic stimulation (TMS), may help resolve this issue. Woo *et al*. applied TMS to participants during visual TOJ^61^. Their results showed that TMS over the right PPC delayed detection of a stimulus presented at the contralateral visual field, whereas TMS over the left PPC did not evoke a significant effect, supporting the involvement of the right PPC in the prior entry effect. Notably, Woo *et al*. used relatively short TMS-onset delays (50-200 ms from the first stimuli in the visual targets), and the effects of TMS on the right PPC peaked at 50 ms after the presentation of the first visual stimuli. These TMS-onset delays were adequate for testing neural causality of the prior entry effect, whereas the delays might be too short to affect the neural function for the determination of the temporal order of stimuli. In Woo *et al*., the average values of the TMS effect on the left PPC increased as the TMS-onset delay increased (from 50 to 200 ms), even though the effect did not eventually reach statistical significance. If longer TMS delays (e.g., > 200 ms) are used, a significant effect on TOJs may appear with TMS applied to the left PPC.

A further difference may exist in multi-sensory tasks. Theoretical opinions have differed between studies dealing with monomodal^23^ and multimodal^28^ tasks. Indeed, in psychophysics, prior experience induces opposite aftereffects on tactile and audiovisual TOJs^4^. Using audiovisual stimuli, different results may be obtained. Some previous neuroimaging studies have reported neural correlates of audiovisual synchrony perception, with respect to cross-modal binding or integration^62^,^63^,^64^. Bushahara *et al*.^63^ reported that audiovisual SJ induced stronger activity in the right PPC, IFG, anterior IC, and left cerebellum, in which the IC exhibited the highest task-specific activity. However, the IC activity was localised to the right and anterior IC, unlike our tactile SJ.

Dahamala *et al*.^64^ investigated brain activity elicited by synchronous and asynchronous audiovisual stimuli sequences presented in a rhythmic stimulation paradigm. In their study, a ternary judgement (synchronous, audio first, or visual first) was used. Their fMRI results during the asynchronous (audio first and visual first) conditions may be compared to those during TOJ, although Dahamala *et al*. did not directly contrast the brain activation levels between the synchronous and asynchronous conditions. The results demonstrated that the auditory, visual, and prefrontal areas were commonly activated during asynchronous and synchronous conditions and that these three areas formed a network with the IPL during the asynchronous conditions. However, the auditory, visual, and prefrontal areas formed a different network with the superior colliculus during the synchronous condition. Moreover, Dahamala’s group^62^ recently reanalysed the selected data and proposed that TOJ involved the right TPJ, dorsolateral prefrontal cortex (DLPFC), and left IPL, whereas synchrony perception (i.e., SJ) involved the left TPJ, DLPFC, and right STG. The results of the study by Dahamala *et al*. suggest that in audiovisual tasks, the difference in neural activity elicited between TOJ and SJ is not observed as a difference in the number or quantity of activated regions.

In our preliminary study using audiovisual stimuli^65^, however, the TOJ > SJ contrast exhibited activation of the left-dominant motor control or general prediction network, similar to the present tactile tasks, whereas there was no activation in the SJ > TOJ contrast. Notably, an fMRI study on motor control demonstrated that a discrete motor task activates more brain regions than a rhythmic motor task^66^. A similar difference in neural activation between discrete and rhythmic tasks may also occur in TOJ because TOJ-specific activity overlaps the motor control network. The discussions over sensory modalities are merely speculative. Further neural evidence is necessary to resolve these issues.

## Methods

### Participants

Sixteen healthy participants (8 men, 8 women, all right-handed; mean age, 25.7 y; range 21–40 y) participated in these experiments. Fourteen participants were naïve subjects, and 2 participants were authors of this manuscript. This study was approved by the ATR-Promotions Review Board Ethics Committee. All participants provided written informed consent. All experiments were performed in accordance with the approved guidelines and regulations.

### Apparatus

During the experiments, the participants laid on their backs with their eyes closed and held a pair of devices using both hands (Fig. 1). Each device consisted of a skin contactor and a response pad.

The heads of the contactors were made of acrylonitrile butadiene styrene resin (2 mm in diameter). The participants placed the ventral pads of their thumbs on the contactor heads (Fig. 1b). Each contact was induced by a bimorph-type piezoelectric actuator (Uchida Dennsi, Tokyo, Japan), and a rectangular voltage pulse (25 V, 4 ms) was applied to produce a small movement (0.3 mm vertically).

Each response pad (Current Designs, Philadelphia, PA, USA) was equipped with two buttons on which the participants placed their index and middle fingers (Fig. 1c). The devices were stabilised so that the participants’ index and middle fingers were positioned vertically to their coronal planes (Fig. 1a).

The participants wore headphones (Hitachi Advanced Systems, Yokohama, Japan), which attenuated the MRI scanning sounds and provided the participants with task instructions.

The timing of the mechanical pulses and the auditory instructions were controlled using Presentation (Neurobehavioral Systems, Berkeley, CA, USA) on a Windows XP computer.

### Tasks

In each trial, the participants were presented with two tactile stimuli, with one stimulus delivered to each hand. The SOAs were pseudo-randomly selected from 11 intervals (−180, −90, −30, −10, −5, 0, +5, +10, +30, +90, and +180 ms; positive values indicate “right preceded left”). The participants performed TOJ or SJ for the tactile stimuli. The TOJ and SJ were performed in a two-alternative forced-choice manner. For TOJ, the participants were required to judge only whether the stimuli were felt right-first or left-first. For SJ, the participants were required to judge only whether the stimuli were simultaneous or non-simultaneous. The participants reported their binary answers by pushing the buttons using the index or middle fingers of one hand.

In our TOJ and SJ, the right and left hand responses were counterbalanced, which was a possible issue in a previous study on visual TOJ^54^. For both tasks, half of the participants used their right hand to report their judgements in the 1st and 2nd sessions and subsequently used their left hand in the 3rd and 4th sessions (4 sessions in total). The remaining participants used their left hand in the 1st and 2nd sessions and subsequently used their right hand in the 3rd and 4th sessions.

To match the methods of response between TOJ and SJ in the present study, we designed the TOJ task to exclude the judgement-response compatibility that was applied to tactile TOJ in a previous fMRI study by Takahashi *et al*.^30^. In this study, the participants pushed a button held by their right hand when the participants judged that their right hand was stimulated last, and vice versa. By contrast, it was impossible to arrange such spatial judgement-response compatibility for SJ; therefore, we designed the method of response for TOJ in a manner that could be used in SJ. Nevertheless, one might argue that the lack of judgement-response compatibility in our TOJ affected TOJ-specific activity. However, this factor does not explain the present results. Notably, the TOJ-specific regions detected herein were included in those detected in Takahashi *et al*.^30^, who used tactile TOJ with judgement-response compatibility.

### Task procedure during fMRI scanning

We used a block design for the task performed in our experiment. Each block was composed of 11 trials, and all 11 of the SOAs appeared within each block. A pair of tactile stimuli was presented every 2.5 s (i.e., each trial lasted 2.5 s). The participants performed only one of the two tasks within each block. At the beginning of each block (2.5 s before the first trial), a female voice recording informed the participants which task to perform during the block in Japanese: “junjo (order)” for TOJ or “douji (simultaneity)” for SJ. Each block was followed by a 15-s rest period. Each session comprised 8 blocks (4 TOJ and 4 SJ blocks). The TOJ and SJ blocks were alternated. A 30-s waiting period was included at the beginning of each session. Each participant performed 4 sessions during the fMRI scanning. That is, each participant performed 11 trials/blocks × 8 blocks/sessions × 4 sessions = 352 trials in total (176 trials for each task) during the scanning.

### Task procedure prior to fMRI scanning (practice sessions)

Prior to scanning, the participants practised the tasks outside the scanner. First, the participants practised one or two blocks (11 trials per block) for each task with each hand to become familiarised with the tasks. Next, the participants performed 2 practice sessions (4 TOJ and 4 SJ blocks per session). The participants responded with their right hand during one session and with their left hand during the other session.

### Imaging

fMRI was performed using a 3T Verio scanner equipped with a 12-channel Head Matrix Coil (Siemens, Munich, Germany). Each session lasted 6.5 min and included a T2*-weight echo planar imaging pulse sequence using the following parameters: repetition time (TR) = 2,500 ms; echo time (TE) = 30 ms; flip angle = 80°; matrix size = 64 × 64; field of view = 192 × 192 mm; 39 ascending 3.2-mm thick slices with a 25% slice gap. After the functional images were acquired, a T1-weighted high-resolution anatomical image was obtained using a magnetisation-prepared rapid-acquisition gradient-echo sequence (TR = 2,250 ms; TE = 3.06 ms; flip angle = 20°; field of view = 256 × 256 mm; voxel size = 1 × 1 × 1 mm).

### Behavioural data analysis

Using the response data, we calculated the response ratios of the binary judgements for TOJ and SJ (Fig. 2a). In previous fMRI studies on TOJs^30^,^54^, the accuracy rates were balanced between TOJs and control tasks. However, this conventional method for balancing the behavioural output between the tasks is inadequate for comparing TOJ and SJ. For TOJ, there are neither correct nor incorrect answers to stimuli with SOA = 0, although SOA = 0 is necessary for SJ. Therefore, we balanced the binary response ratios between TOJ and SJ. A block design was adopted in our study. Across the blocks, sensory inputs and motor outputs were thus balanced between TOJ and SJ.

Moreover, the judgement data were sorted according to SOA, and the judgement proportions were fit, as a function of SOA, to a cumulative Gaussian function for TOJ (Fig. 2b) and to a Gaussian frequency function for SJ (Fig. 2c) using the maximum likelihood method. From the fitted Gaussian functions, we obtained the PSS (mean) and σ (SD) for each task. Instead of the σ, the just noticeable difference (JND; half of the SOA difference between 0.75 and 0.25 points of the judgement ratio at the sigmoidal psychometric function) has also been used as an index of time sensitivity in previous TOJ studies^5^,^9^. However, the σ value can be compared directly between TOJ and SJ^24^. In this study, the participants exhibited greater σ values for TOJ than for SJ (see *Behavioural results*). The greater σ values for TOJ than for SJ are consistent with the psychophysical observations in previous studies using tactile^24^, audio^22^, and visual^23^ stimuli. Thus, a difference in time sensitivity inevitably occurs between monomodal TOJ and SJ. Accordingly, we excluded the effect of the σ difference between the tasks at the stage of the brain imaging analysis (see *fMRI data analysis*).

### fMRI data analysis

Imaging and statistical analyses were performed using the statistical parametric mapping package SPM8 (http://www.fil.ion.ucl.ac.uk/spm). Functional images of each run (i.e., session) were realigned using the first scan as a reference to correct for head movements. The T1 anatomical image was preprocessed using the intensity inhomogeneity correction. The T1 anatomical images were coregistered to the first scan of the functional images. Subsequently, the coregistered T1 anatomical images were normalised to a standard T1-template image defined by the Montreal Neurological Institute (MNI), which involved linear and nonlinear three-dimensional transformations. The normalisation parameters were subsequently applied to each of the functional images. Finally, the spatially normalised functional images were resampled to a voxel size of 2 × 2 × 2 mm and smoothed using an isotopic Gaussian kernel of 8-mm full-width at half-maximum to compensate for anatomical variability amongst the participants. The reduction in the voxel size by subsampling and spatial smoothing ensures a good lattice approximation, which is necessary for correcting the statistical inference using the random field theory^67^.

We used a random effects model for the statistical analysis. First, we performed a single-participant analysis. The task-related neural activity relative to the baseline resting periods was modelled using a boxcar function and convolved with a canonical haemodynamic response function. We used a high-pass filter with a discrete cosine basis function and a cut-off period of 128 s to eliminate artefactual low-frequency trends. Serial autocorrelation, assuming a first-order autoregressive model, was estimated using the pooled active voxels with a restricted maximum likelihood procedure, and the estimates were used to whiten the data and design matrices.

The statistical model included two conditions: TOJ and SJ. T-contrasts were set to perform the following comparisons: TOJ > SJ; SJ > TOJ; TOJ > rest; and SJ > rest. The contrast images were generated for each subject and then entered into a one-sample *t*-test to create a random effect SPM. The SPM{*T*} was transformed into normal distribution units SPM{*Z*}. Significantly activated voxels were identified using a threshold of *P* < 0.001 uncorrected at the voxel level (*Z* = 3.09) and *P* < 0.05 FWE-corrected at the cluster level^41^.

In the TOJ > SJ and SJ > TOJ contrasts, we excluded the effect of the σ difference between the tasks, according to the conventions that the behavioural difference between the tasks should be compensated for in the fMRI contrasts^39^,^40^. The σ difference for each subject was regressed out as a covariate of no interest in these contrasts. Furthermore, the voxels that were correlated with the σ difference (*P* < 0.05 uncorrected at the voxel level) were exclusively masked in the present study. In addition, to avoid false activations as a result of deactivations in the contrasting conditions, the TOJ > SJ and SJ > TOJ contrasts were inclusively masked with the TOJ > rest and SJ > rest contrasts (*P* < 0.05 uncorrected at the voxel level), respectively.

We labelled the brain regions and Brodmann areas (BAs) using Talairach Client (ver. 2.4.3; http://www.talairach.org/client.html) after transforming the MNI coordinates of the peak activations to Talairach coordinates using icbm2tal (http://www.brainmap.org/icbm2tal/). We also assessed the labels using the Talairach coordinates transformed by mni2tal (http://medicine.yale.edu/bioimaging/suite/mni2tal/). When discrepancies occurred between the labels using icbm2tal and mni2tal or when there was no output from the Talairach Client, we adjusted or compensated for the labels using the SPM Anatomical Toolbox^68^ or MRIcron (www. mricro.com).

## Additional Information

**How to cite this article**: Miyazaki, M. *et al*. Dissociating the neural correlates of tactile temporal order and simultaneity judgements. *Sci. Rep*. **6**, 23323; doi: 10.1038/srep23323 (2016).

## Supplementary Material

Supplementary Information
