# Supplementary material for: Dissociating the neural correlates of tactile temporal order and simultaneity judgements
Source: Sci Rep. 2016 Apr 11;6:23323. doi: 10.1038/srep23323 (PMC4827393; doi:10.1038/srep23323)
Supplement: Supplementary Information [file srep23323-s1.pdf]

Supplementary information

Dissociating the neural correlates of tactile temporal order and simultaneity judgements

Makoto Miyazaki[1,2*], Hiroshi Kadota[2], Kozue S Matsuzaki[2], Shigeki Takeuchi[3], Hirofumi Sekiguchi[3], Takuo Aoyama[4], Takanori Kochiyama[5]

1. Department of Computer Science, Faculty of Informatics, Shizuoka University
2. Research Institute, Kochi University of Technology
3. Faculty of Business and Information Sciences, Jobu University
4. Research Institute for Time Studies, Yamaguchi University
5. ATR Brain Activity Imaging Center

***Corresponding author:**

Makoto Miyazaki, Faculty of Informatics, Shizuoka University, 3-5-1 Johoku, Naka-ku, Hamamatsu, Shizuoka 432-8011, Japan

< miyazaki-makoto@inf.shizuoka.ac.jp >

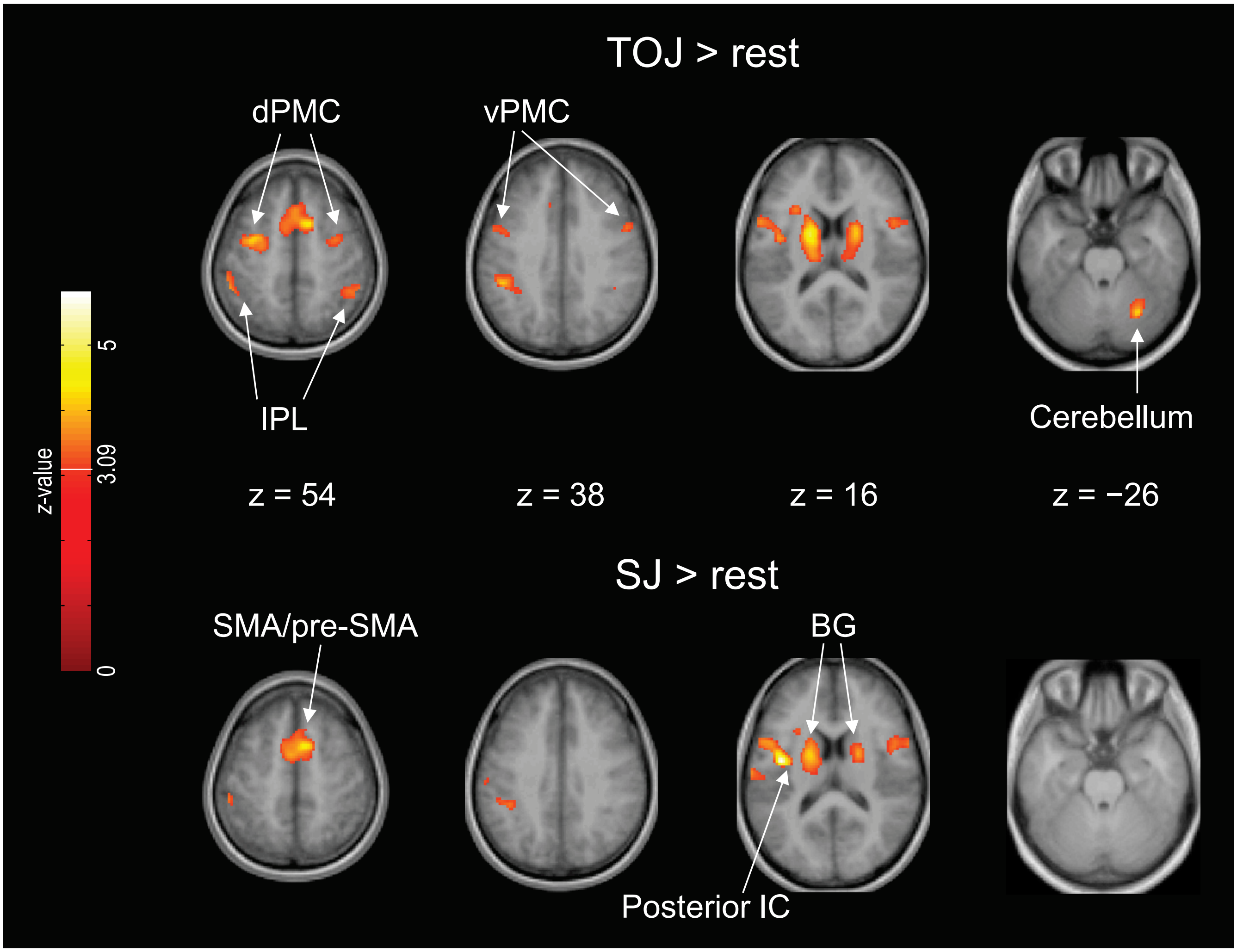

Figure S1. Brain regions that were more strongly activated during TOJ and SJ than during the baseline resting periods. The results are from a 16-participant group analysis. Significantly activated voxels were identified using a significance threshold of $P < 0.001$ uncorrected at the voxel level ($Z = 3.09$) and $P < 0.05$ FWE-corrected at the cluster level. dPMC, dorsal premotor cortex; vPMC, ventral premotor cortex; IPL, inferior parietal lobule; SMA, supplementary motor area; IC, insular cortex; BG, basal ganglia.

Table S1. Regions exhibiting greater activity during TOJ than during SJ. The activated voxels were identified using a significance threshold of $P < 0.001$ uncorrected at the voxel level ($Z = 3.09$) and $P < 0.05$ FWE-corrected at the cluster level. The clusters shown underneath the dashed line were detected using the liberal threshold at the cluster level ($P < 0.05$ uncorrected) without changing the threshold at the voxel level ($P < 0.001$ uncorrected). In this contrast, the σ difference between tasks was modelled as a covariate of no interest, and the voxels that correlated with the σ difference ($P < 0.05$ uncorrected at the voxel level) were exclusively masked. This contrast was also inclusively masked with the TOJ > rest contrast ($P < 0.05$ uncorrected at the voxel level).

						MNI Coodinates		
Cluster#	Size (voxel)	z-score	L/R	Region	BA	x	y	z
1	540	4.84	L	Precentral Gyrus	6	-32	-4	54
		4.81	L	Precentral Gyrus	6	-32	-2	58
		4.34	L	Cingulate Gyrus/Middle Frontal Gyrus	6	-24	-16	46
		4.33	L	Precentral Gyrus/Superior Frontal Gyrus	6	-24	-10	54
2	221	4.80	L	Precentral Gyrus	6	-48	2	36
3	199	4.31	R	Middle Frontal Gyrus	6	28	-4	54
		4.04	R	Medial Frontal Gyrus/Middle Frontal Gyrus	6	22	-8	50
4	529	4.25	L	Superior Parietal Lobule	7	-30	-62	48
		4.00	L	Superior Parietal Lobule	7	-30	-64	58
		3.89	L	Superior Temporal Gyrus/Inferior Parietal Lobule	39/40	-30	-52	40
		3.84	L	Inferior Parietal Lobule	7	-34	-54	50
		3.43	L	Inferior Parietal Lobule	40	-44	-50	56
		3.36	L	Inferior Parietal Lobule	40	-46	-46	50
		3.26	L	Inferior Parietal Lobule	40	-48	-42	40
5	240	3.89	R	Thalamus		10	-12	0
		3.84	R	Thalamus/Subthalamic Nucleus		8	-14	-6
		3.80	R	Thalamus		12	-8	2
		3.65	R	Thalamus		2	-12	4
		3.60	L	Thalamus		-6	-12	-2
		3.58	L	Thalamus		-6	-14	2
		3.36	L	Thalamus		-10	-6	6
6	140	3.90	R	Cerebellum		32	-68	-28
		3.87	R	Cerebellum		28	-60	-32
		3.61	R	Cerebellum		36	-62	-32
7	171	3.81	L	Insula	13	-34	28	6
		3.71	L	Insula	13	-34	24	8
		3.63	L	Claustrum		-28	20	8
		3.61	L	Claustrum		-32	14	0
8	86	3.80	R	Cerebellum		26	-68	-48
		3.76	R	Cerebellum		22	-66	-48
		3.65	R	Cerebellum		26	-60	-44
9	104	3.72	R	Medial Frontal Gyrus/Superior Frontal Gyrus	6	12	8	58
		3.49	L	Medial Frontal Gyrus/Superior Frontal Gyrus	6	-8	12	52
		3.37	L	Superior Frontal Gyrus	6	-2	14	54

Table S2. Regions exhibiting greater activity during SJ than during TOJ. The activated voxels were identified using a significance threshold of $P < 0.001$ uncorrected at the voxel level ($Z = 3.09$) and $P < 0.05$ FWE-corrected at the cluster level. The clusters shown underneath the dashed line were detected using the liberal extent threshold at the cluster level ($P < 0.05$ uncorrected) without changing the threshold at the voxel level ($P < 0.001$ uncorrected). In this contrast, the σ difference between tasks was modelled as a covariate of no interest, and the voxels that correlated with the σ difference ($P < 0.05$ uncorrected at the voxel level) were exclusively masked. This contrast was also inclusively masked with the SJ > rest contrast ($P < 0.05$ uncorrected at the voxel level).

						MNI Coodinates		
Cluster#	Size (voxel)	z-score	L/R	Region	BA	x	y	z
1	259	4.03	L	Insula	13	-42	0	8
		4.01	L	Superior Temporal Gyrus	22	-48	2	-2
		3.98	L	Insula	13	-46	-2	4
		3.92	L	Insula/Rolandic Operculum	13	-48	-6	8
		3.89	L	Insula	13	-40	-10	18
2	120	3.62	R	Insula	13	44	-2	10
		3.60	R	Insula/Superior Temporal Gyrus	13/22	48	2	4
		3.51	R	Insula	13	40	2	12
		3.47	R	Insula	13	40	-4	18
		3.38	R	Insula	13	40	0	0

Table S3. Regions exhibiting greater activity during TOJ than during the baseline resting periods. The activated voxels were identified using a significance threshold of $P < 0.001$ uncorrected at the voxel level ($Z = 3.09$) and $P < 0.05$ FWE-corrected at the cluster level.

						MNI Coodinates		
Cluster#	Size (voxel)	z-score	L/R	Region	BA	x	y	z
1	1293	5.51	R	Medial Frontal Gyrus/Superior Frontal Gyrus	6	10	6	60
		4.81	L	Medial Frontal Gyrus	6	-10	10	50
		4.49	L	Medial Frontal Gyrus	6	-8	-2	60
		4.11	L	Medial Frontal Gyrus/Superior Medial Gyrus	32	-8	20	42
		4.04		Superior Frontal Gyrus	6/8	0	16	54
		3.80	R	Superior Frontal Gyrus	32	8	22	48
2	5745	5.45	L	Claustrum		-30	20	2
		5.21	L	Caudate/Putamen		-18	0	16
		5.07	L	Thalamus		-12	-12	2
		5.05	L	Thalamus		-14	-12	8
		5.03	L	Caudate		-14	6	8
		5.02	R	Insula		34	20	2
		4.93	R	Thalamus		12	0	8
		4.89	R	Thalamus		12	-16	4
		4.89	L	Thalamus		-10	-4	8
		4.88	L	Thalamus		-10	0	6
		4.82	L	Putamen		-26	2	-2
		4.73	L	Insula	13	-38	16	6
		4.62	R	Caudate/Putamen		18	2	14
		4.54	R	Putamen		20	4	2
		4.36	R	Putamen		28	2	-2
		4.33	L	Inferior Frontal Gyrus	9/44	-56	8	24
		4.17	L	Red Nucleus		-2	-26	-2
		4.16	L	Precentral Gyrus	6	-48	0	34
		4.10	L	Insula	13	-42	-4	16
		4.07	R	Red Nucleus		10	-22	-8
		3.78	R	Inferior Frontal Gyrus	9/44	50	8	16
		3.76	L	Precentral Gyrus	44	-54	12	4
3	453	4.61	L	Precentral Gyrus	6	-34	-6	54
4	290	4.60	R	Cerebellum		28	-64	-26
		4.46	R	Cerebellum		32	-56	-30
		3.50	R	Cerebellum		38	-44	-34
5	581	4.51	L	Inferior Parietal Lobule	40	-48	-40	38
		4.10	L	Inferior Parietal Lobule	40	-52	-44	54
		3.89	L	Inferior Parietal Lobule	40	-56	-38	50
		3.74	L	Inferior Parietal Lobule	40	-46	-50	56
		3.66	L	Inferior Parietal Lobule	40	-36	-48	44
6	422	4.47	R	Inferior Parietal Lobule	40	42	-46	48
		4.21	R	Inferior Parietal Lobule	40	54	-46	50
		3.99	R	Inferior Parietal Lobule	40	48	-48	56
		3.82	R	Inferior Parietal Lobule	40	58	-34	50
7	298	4.14	R	Precentral Gyrus	6	32	-8	50
		3.95	R	Precentral Gyrus	6	54	4	42
		3.47	R	Precentral Gyrus	6	42	-6	48

Table S4. Regions exhibiting greater activity during SJ than during the baseline resting periods. The activated voxels were identified using a significance threshold of $P < 0.001$ uncorrected at the voxel level ($Z = 3.09$) and $P < 0.05$ FWE-corrected at the cluster level.

						MNI Coodinates		
Cluster#	Size (voxel)	z-score	L/R	Region	BA	x	y	z
1	2690	5.82	L	Insula	13	-42	-6	16
		4.99	L	Claustrum		-28	22	6
		4.59	L	Putamen		-18	4	10
		4.58	L	Putamen		-18	8	4
		4.57	L	Caudate/Putamen		-18	-2	16
		4.44	L	Putamen		-26	0	-2
		4.44	L	Putamen		-24	0	2
		4.25	L	Inferior Frontal Gyrus	44	-50	8	20
		4.19	L	Inferior Frontal Gyrus	9/44	-54	8	24
		4.10	L	Insula	13	-40	16	8
		4.09	L	Inferior Frontal Gyrus	44	-56	8	12
		3.89	L	Thalamus		-12	-20	8
		3.53	L	Thalamus/Subthalamic Nucleus		-14	-16	-4
2	1132	5.09	R	Medial Frontal Gyrus/Superior Frontal Gyrus	6	6	6	58
		5.07	L	Medial Frontal Gyrus	6	-6	-2	60
		4.63	L	Medial Frontal Gyrus	6	-8	8	50
		4.12	R	Medial Frontal Gyrus	6	10	-4	62
		4.07	R	Superior Frontal Gyrus	6	14	0	74
3	1482	4.57	R	Pallidum		14	4	2
		4.40	R	Putamen		20	4	2
		4.36	R	Caudate/Putamen		20	0	14
		4.31	R	Insula	13	32	20	8
		4.03	R	Inferior Frontal Gyrus	9/44	60	8	20
		3.82	R	Inferior Frontal Gyrus	9/44	50	8	14
		3.76	R	Insula/Rolandic Operculum	13/44	48	4	14
		3.49	R	Insula	13	42	12	8
4	578	4.33	L	Postcentral Gyrus	1/3	-66	-14	24
		3.75	L	Supramarginal Gyrus	40	-42	-40	38
		3.73	L	Inferior Parietal Lobule	40	-56	-36	50
		3.72	L	Inferior Parietal Lobule	40	-54	-36	54
		3.58	L	Postcentral Gyrus	2	-56	-24	48
		3.55	L	Postcentral Gyrus	3	-58	-20	44
		3.49	L	Inferior Parietal Lobule/ Supramarginal Gyrus	40	-52	-32	26
		3.48	L	Inferior Parietal Lobule	40	-50	-38	38
		3.47	L	Inferior Parietal Lobule	40/2	-56	-28	50

Table S5. Regions commonly activated during TOJ and SJ. The activated voxels were identified by inclusively masking the TOJ > rest contrast [$P < 0.001$ uncorrected at the voxel level ($Z = 3.09$) and $P < 0.05$ FWE-corrected at the cluster level] with the SJ > rest contrast [$P < 0.001$ uncorrected at the voxel level ($Z = 3.09$) and $P < 0.05$ FWE-corrected at the cluster level]. Note that the use of right and left hands for responding during the tasks was counterbalanced in the present study. Accordingly, brain activation of the relatively late stage for motor response processing (e.g., the left and right primary sensorimotor cortices) was cancelled out in the contrasts between the tasks and the baseline resting periods.

						MNI Coodinates		
Cluster#	Size (voxel)	z-score	L/R	Region	BA	x	y	z
1	914	5.51	R	Medial Frontal Gyrus/Superior Frontal Gyrus	6	10	6	60
		4.81	L	Medial Frontal Gyrus	6	-10	10	50
		4.49	L	Medial Frontal Gyrus	6	-8	-2	60
		4.04		Superior Frontal Gyrus	6/8	0	16	54
		3.80	R	Superior Frontal Gyrus/Medial Frontal Gyrus	6/8	8	20	50
2	2305	5.45	L	Claustrum		-30	20	2
		5.21	L	Caudate/Putamen		-18	0	16
		5.07	L	Thalamus		-12	-12	2
		5.05	L	Thalamus		-14	-12	8
		5.03	L	Caudate		-14	6	8
		4.89	L	Thalamus		-10	-4	8
		4.88	L	Thalamus		-10	0	6
		4.82	L	Putamen		-26	2	-2
		4.73	L	Insula	13	-38	16	6
		4.33	L	Inferior Frontal Gyrus	9/44	-56	8	24
		4.10	L	Insula	13	-42	-4	16
		3.76	L	Precentral Gyrus	44	-54	12	4
3	1190	5.02	R	Insula		34	20	2
		4.93	R	Thalamus		12	0	8
		4.62	R	Caudate/Putamen		18	2	14
		4.54	R	Putamen		20	4	2
		4.36	R	Putamen		28	2	-2
		3.90	R	Insula	13	42	16	6
		3.78	R	Inferior Frontal Gyrus	9/44	50	8	16
